# Enabling Robust N‐Type Perovskite Field‐Effect Transistors Through an TiO_2_ Interlayer Strategy

**DOI:** 10.1002/advs.202516610

**Published:** 2025-10-07

**Authors:** Jiangnan Xia, Xincan Qiu, Ping‐An Chen, Yu Liu, Jiaqi Ding, Yu Zhang, Huan Wei, Zhenqi Gong, Chengyuan Peng, Wenpei Shi, Shuanglong Wang, Chen Chen, Yuanyuan Hu

**Affiliations:** ^1^ International Science and Technology Innovation Cooperation Base for Advanced Display Technologies of Hunan Province College of Semiconductors (College of Integrated Circuits) Hunan University Changsha 410082 China; ^2^ Key Laboratory for Micro/Nano Optoelectronic Devices of Ministry of Education School of Physics and Electronics Hunan University Changsha 410082 China; ^3^ Key Laboratory of Hunan Province for 3D Scene Visualization and Intelligence Education School of Electronic Information Hunan First Normal University Changsha 410205 China; ^4^ Science and Technology on Advanced Ceramic Fibers and Composites Laboratory College of Aerospace Science and Engineering National University of Defense Technology Changsha 410000 China; ^5^ Department of Applied Physics The Hong Kong Polytechnic University Hong Kong 999077 China

**Keywords:** dion‐jacobson (DJ) phase perovskite, field‐effect transistor, metal halide perovskites, photodetector, TiO_2_ interlayer

## Abstract

Metal halide perovskites (MHPs) show tremendous potential for field‐effect transistors (FETs), but N‐type Pbbased MHP FETs have been hindered by critical challenges, including high defect densities, ion migration, and poor reproducibility. In this work, a simple yet powerful ultrathin TiO_2_ interlayer strategy is introduced that fundamentally transforms the fabrication of Pb‐based MHP FETs. By pre‐depositing an ultrathin TiO_2_ layer before perovskite film deposition, reproducible and operationally stable MAPbI_3_ FETs with remarkable performance are achieved. Comprehensive characterizations reveal that the TiO_2_ interlayer enhances precursor wetting, promotes larger and more uniform grain formation, reduces defect density, and effectively suppresses non‐radiative recombination and ion migration. The universality of this approach is demonstrated by successfully extending it to 2D Dion‐Jacobson phase perovskites, including PDAPbI_4_ and its derivatives. The fabricated devices exhibit excellent electrical characteristics, including high on/off ratios, low hysteresis, and impressive stability. As a proof of concept, a complementary inverter is constructed using perovskite‐only components, showcasing the potential for integrated logic circuits. This work provides a robust fabrication method for high‐performance Pb‐based perovskite FETs with broad applicability.

## Graphical abstract

An ultrathin TiO_2_ interlayer strategy is developed for fabricating high‐performance, lead‐based, N‐type perovskite field‐effect transistors. This method improves film quality, reduces defects, and suppresses ion migration, enabling reproducible and stable devices. The strategy is successfully applied to both 3D (MAPbI_3_) and 2D Dion‐Jacobson phase perovskites, demonstrating its broad applicability and paving the way for perovskite‐based circuits.

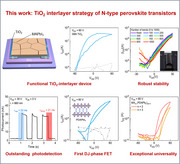

## 1 Introduction

Metal halide perovskite (MHP) semiconductors have emerged as highly promising candidates for field‐effect transistors (FETs) applications, owing to their exceptional solution processability and high intrinsic charge carrier mobility.^[^ ^1^, ^2^, ^3^ ^]^ In recent years, significant advancements have been achieved in P‐type FETs based on Sn‐based MHP semiconductors, with hole mobilities exceeding 50 cm^2^/V·s, demonstrating remarkable performance potential.^[^ ^4^, ^5^, ^6^, ^7^ ^]^ Despite significant advancements in the development of P‐type MHP FETs, their N‐type counterparts, particularly Pb‐based MHP FETs, have exhibited markedly slower progress.

Pb‐based MHP semiconductors, known for their ambipolar charge transport characteristics, have been widely utilized in N‐type FET devices. However, the performance of Pb‐based MHP FETs is often compromised by inherent material challenges, including high carrier defect densities, structural phase transitions, and ion migration, which collectively manifest in severely hysteretic transfer characteristics and, in extreme cases, complete loss of gate modulation capability.^[^ ^8^, ^9^ ^]^ Although MAPbI_3_ (MA = Methylammonium) FETs capable of operating at room temperature have been reported through strategies such as solvent passivation,^[^ ^10^ ^]^ additive engineering,^[^ ^11^, ^12^ ^]^ and single‐crystal synthesis,^[^ ^13^, ^14^ ^]^ these approaches often involve intricate experimental procedures and high operational complexity, limiting their reproducibility. This limitation significantly hinders the progress in optimizing the performance of Pb‐based MHP FETs. Therefore, the development of a simple, efficient, and highly reproducible fabrication method is of paramount importance for advancing the performance of Pb‐based MHP FET devices.

In this work, we propose a simple, robust interlayer strategy for fabricating Pb‐based FETs. Using MAPbI_3_ as a model system, an ultrathin TiO_2_ interlayer is pre‐deposited on the substrate prior to perovskite film formation. This modification yields reproducible and operationally stable MAPbI_3_ FETs. Structural, morphological, and electrical characterizations reveal that the adoption of the TiO_2_ interlayer benefits the wetting of the perovskite precursor solution, improving the film morphology, effectively reducing defects in the MAPbI_3_, suppressing non‐radiative recombination, and dramatically mitigating ion migration, all of which lead to the enhancement of device performance. We further demonstrate the generality of the approach: it is effective not only for 3D Pb‐based perovskites but also for other Pb‐perovskite compositions. Notably, using this method, we have, for the first time, fabricated large‐area thin‐film FET arrays based on a 2D Dion–Jacobson (DJ) phase Pb perovskite (PDAPbI_4_) that operate at room temperature. Moreover, we built a complementary inverter entirely from perovskite FETs by combining an N‐type PDAPbI_4_ device with a P‐type PEA_2_SnI_4_ device, demonstrating basic circuit functionality. Our work therefore provides a simple, effective, and reproducible fabrication route that lays a practical foundation for advancing Pb‐based N‐type MHP FETs and their integration into functional circuits.

## 2 Results and Discussion

### 2.1 TiO_2_ Interlayer Strategy for MAPbI_3_ FET Fabrication

Traditional fabrication of MAPbI_3_ FETs faces two major challenges that hinder room‐temperature operation and reproducibility: first, the requirement for complex defect passivation processes to improve film quality,^[^ ^10^, ^11^, ^12^ ^]^ and second, the inherent limitations imposed by anti‐solvent crystallization techniques on fabrication yield.^[^ ^15^ ^]^ Consequently, the achievement of N‐type MAPbI_3_ FETs that can function well at room temperature remains a formidable challenge, significantly impeding the advancement of N‐type perovskite FETs.

**Figure 1:**
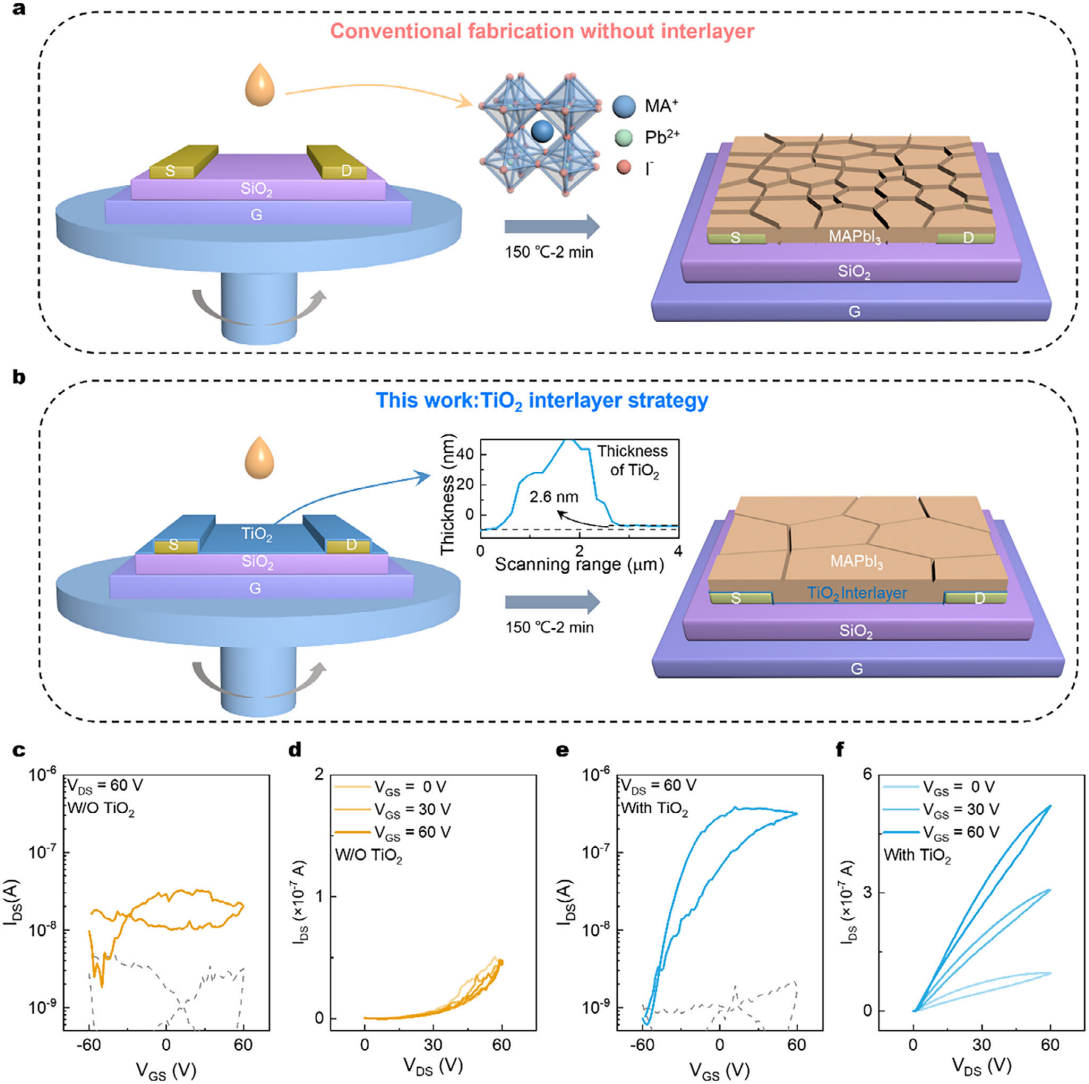
Enhanced device performance via TiO_2_ interlayer engineering. Schematic of the MAPbI_3_ FET device structure a) without and b) with TiO_2_ interlayer. The c) transfer and d) output characteristic curves of the MAPbI_3_ FET without TiO_2_ interlayer. The e) transfer and f) output characteristic curves of the MAPbI_3_ FET with TiO_2_ interlayer.

We streamlined the fabrication process of reference MAPbI_3_ FETs. As illustrated in **Figure** **1**a, the MAPbI_3_ precursor solution was prepared using lead acetate trihydrate (PbAc_2_) and MAI as starting materials, then spin‐coated directly onto pre‐patterned source/drain (S/D) electrodes on a SiO_2_/Si substrate, forming the MAPbI_3_ active layer in a single‐step, anti‐solvent‐free process. Figure S1 (Supporting Information) demonstrates that the MAPbI_3_ films prepared without anti‐solvents exhibit excellent crystallinity; the primary X‐ray diffraction (XRD) diffraction peaks at 14.6°, 28.9°, and 43.6° correspond to the (110), (220), and (314) planes of MAPbI_3_, respectively. The UV‐vis absorption spectrum of MAPbI_3_ shows a steep absorption edge at 760 nm, and the steady‐state photoluminescence (PL) spectrum peaks at 765 nm, corresponding to an optical bandgap of ≈1.55 eV. These results are consistent with previous studies.^[^ ^16^, ^17^ ^]^ Despite the high film quality, the fabricated bottom‐gate bottom‐contact (BGBC) FETs show no gate modulation. As shown in Figure 1c,d, the transfer and output characteristic curves demonstrate a complete loss of gate modulation, with the channel current (I_DS_) showing no response to gate voltage variations. This absence of field‐effect modulation is likely caused by the intrinsic ionic migration and defect‐related trapping in the MAPbI_3_ films, which screens the gate field and prevents accumulation/depletion of mobile carriers in the channel.

Notably, when an ultrathin TiO_2_ interlayer (≈2.6 nm) was pre‐deposited prior to spin‐coating the MAPbI_3_ active layer (Figure 1b; Figure S3, Supporting Information), the MAPbI_3_ FETs performance exhibited remarkable enhancement. As shown in Figure 1e,f, MAPbI_3_ FET devices with the TiO_2_ interlayer demonstrated several orders of magnitude improvement in both on/off ratio and on‐state current under identical gate voltage sweeps. The output characteristic curve now clearly exhibited gate‐voltage modulation, displaying N‐type FET behavior.

To rule out the possibility that the channel current originates from the TiO_2_ interlayer itself, we fabricated BGBC FETs with containing TiO_2_ only in the channel. As shown in Figure S4 (Supporting Information), these TiO_2_‐only FETs exhibit channel currents below 10^−9^ A across the entire gate voltage sweep range, demonstrating insulating behavior. This result confirms that the measured current in the MAPbI_3_ FET originates from charge transport in the MAPbI_3_ active layer rather than conduction through the TiO_2_.^[^ ^18^ ^]^

### 2.2 Top‐Gate MAPbI_3_ FET Fabrication via TiO_2_ Interlayer

**Figure 2:**
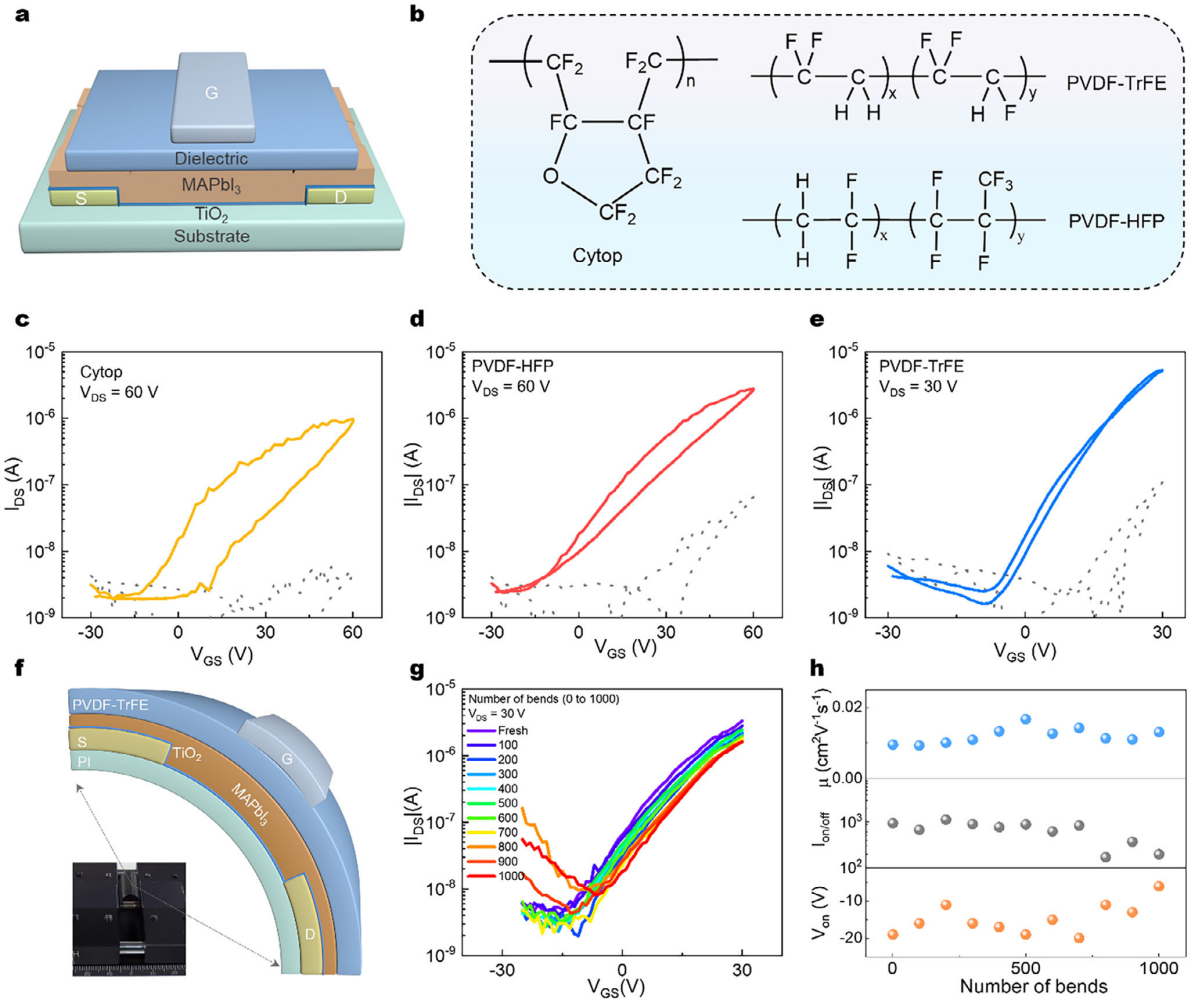
Top‐gate MAPbI_3_ FETs fabricated via TiO_2_ interlayer strategy. a) Schematic diagram of the top gate structure MAPbI_3_ FET through TiO_2_ interlayer. b) Chemical structures of the three dielectric materials: Cytop, PVDF‐HFP, and PVDF‐TrFE. Transfer characteristics of top gate MAPbI_3_ FETs using c) Cytop, d) PVDF‐HFP, and e) PVDF‐TrFE as dielectric layers, respectively. f) Schematic diagram of the flexible MAPbI_3_ FET structure. Inset shows the illustration of the bending test for the flexible device. The bending radius is ≈ 7 mm. g) Changes in the transfer characteristic curves and h) corresponding performance parameters of the flexible device after 1000 bending cycles.

We fabricated top‐gate bottom‐contact (TGBC) structure MAPbI_3_ FETs using the TiO_2_ interlayer. As illustrated in **Figure** **2**a, we first selected common polymer dielectric Cytop as the dielectric for TGBC structure MAPbI_3_ FETs with TiO_2_ interlayer (Figure 2b). The transfer characteristics curves of the Cytop‐based MAPbI_3_ FETs are presented in Figure 2c, which exhibit typical N‐channel transistor characteristics. Importantly, in such TGBC devices, the channel is formed at the top dielectric/MAPbI_3_ interface rather than at the bottom TiO_2_/MAPbI_3_ interface,^[^ ^19^ ^]^ and thus the operation of the device again suggests the charge transport in the MAPbI_3_ rather than in TiO_2_.

We subsequently employed ferroelectric polymers PVDF‐HFP and PVDF‐TrFE as dielectric materials for the TGBC devices. As shown in Figure 2d,e, devices with PVDF‐HFP and PVDF‐TrFE dielectrics exhibited superior performance in terms of smaller hysteresis than the Cytop devices, which is consistent with previous findings that ferroelectric dielectric is beneficial to Pb‐perovskite devices.^[^ ^20^ ^]^ In particular, MAPbI_3_ FETs with PVDF‐TrFE dielectrics demonstrated nearly hysteresis‐free operation, with on/off ratios approaching 10^4^ and low turn‐on voltages. Notably, the TGBC MAPbI_3_ FETs with PVDF‐TrFE dielectrics also display decent bias‐stress stability: on‐currents exhibit only a slight decrease with prolonged bias stress (Figure S5, Supporting Information).

Besides, we replaced the glass substrate with a PI substrate to fabricate a flexible TGBC MAPbI_3_ FET device (Figure 2f), marking the first room‐temperature operational flexible Pb‐based MHP FET. After 1000 bending cycles, the transfer characteristics remain largely unchanged, with only a modest increase in off‐state current appearing after ≈700 cycles, likely due to structural damage in the PVDF‐TrFE dielectric layer during bending. The stable trends in on/off ratio, threshold voltage, and mobility after 1000 cycles indicate excellent device stability (Figure 2g,h).

**Figure 3:**
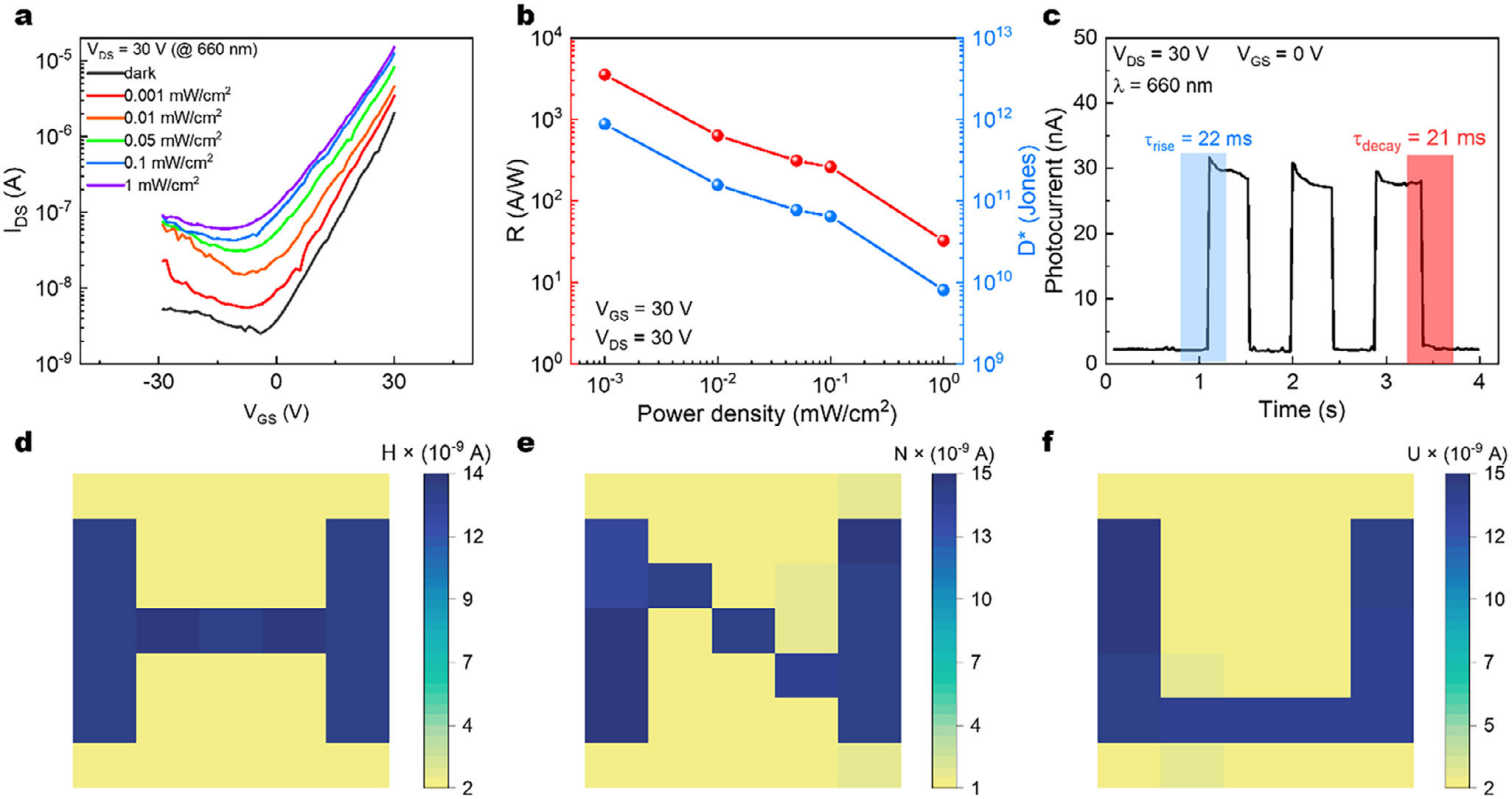
Photodetection performance of the TGBC MAPbI_3_ FET with TiO_2_ interlayer. a) Transfer characteristics of interlayer based MAPbI_3_ device under various illumination power intensities (𝜆 = 660 nm, V_DS_ = 30 V). b) Responsivity and detectivity values of the interlayer‐based MAPbI_3_ device as a function of power intensity at V_GS_ of 30 V. c) The time‐dependent photocurrent measurements of the interlayer‐based MAPbI_3_ device under 660 nm light illumination. d) “H”, e) “N”, and f) “U” shaped mask (660 nm, power density = 0.01 mW cm^−2^, V_GS_ = 0 V).

Following the development of this high‐performance, room‐temperature PVDF‐TrFE‐based MAPbI_3_ FET, we evaluated its photodetection performance at 660 nm. **Figure** **3**a displays the transfer characteristics under 660 nm illumination at varying optical power densities. The device exhibits pronounced photogating and photoconductive effects even at a low light intensity of 0.001 mW cm^−2^. Figure 3b illustrates the dependence of responsivity (R) and specific detectivity (D^*^) on illumination intensity at gate voltage of 30 V. The peak values reach 3 × 10^3^ A/W for R and 10^12^ Jones for D^*^, demonstrating exceptional red‐light detection capabilities. The dynamic response was quantified by measuring the photocurrent rise and decay times, yielding values of 22 ms and 21 ms, respectively (Figure 3c). To demonstrate practical imaging potential, we fabricated a device array consisting of 5 × 5 devices. Leveraging the remarkable of the device to 660 nm light, the MAPbI_3_ FET array successfully discriminates the letters “H,” “N,” and “U” with high contrast (Figure 3d,e), highlighting its promising application in photo imaging technologies.

### 2.3 Understanding the Role of TiO_2_ Interlayer

**Figure 4:**
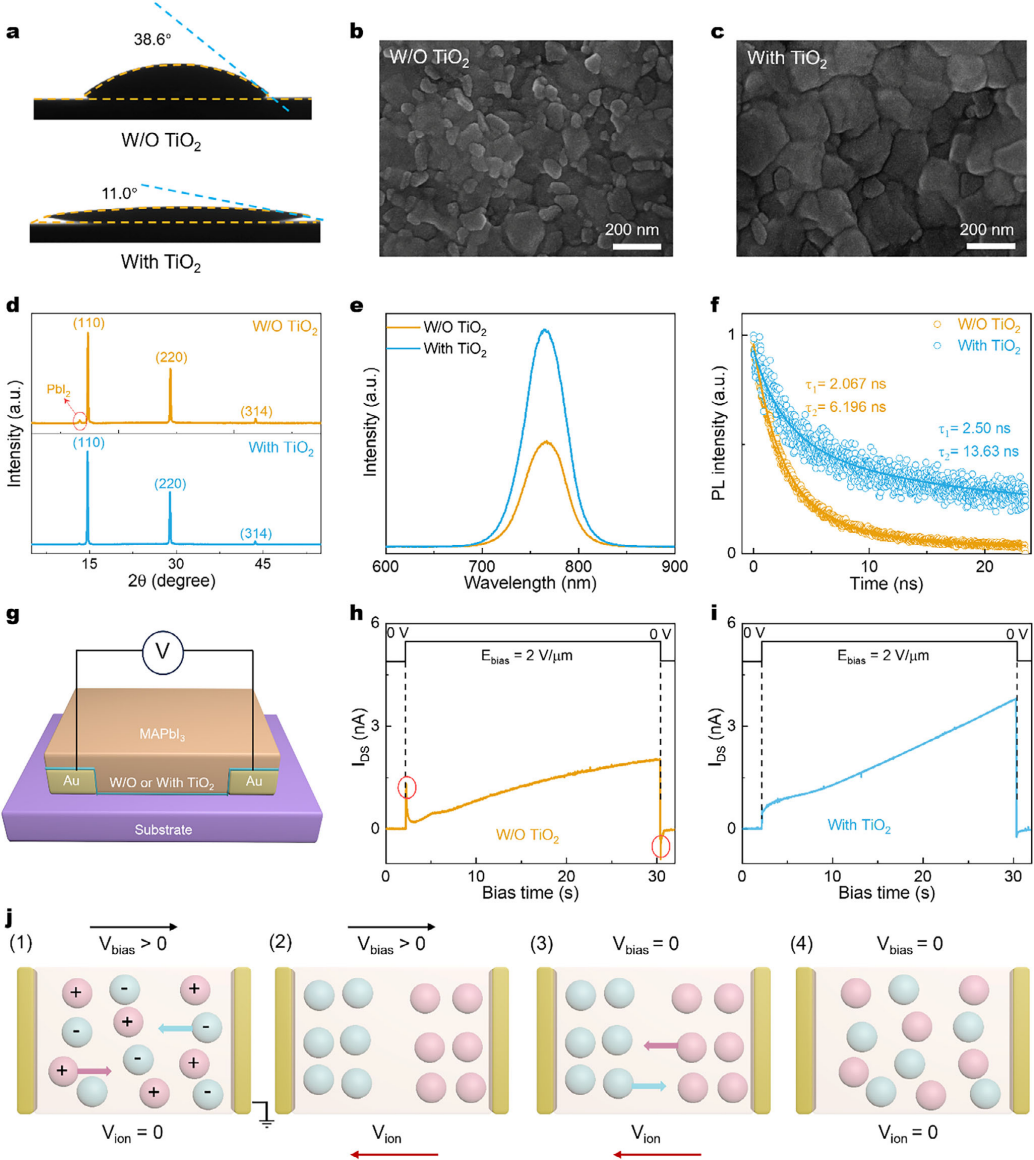
Impact of TiO_2_ on MAPbI_3_ films. a) Contact‐angle measurements of the MAPbI_3_ precursor on substrates without and with the ultrathin TiO_2_ interlayer. b, c) SEM micrographs showing surface morphology of MAPbI_3_ films prepared (b) directly on the bare substrate and (c) on the TiO_2_‐coated substrate. d) XRD patterns comparing the crystallinity of MAPbI_3_ films with and without the TiO_2_ interlayer. e) Steady‐state PL spectra and f) time‐resolved PL decay traces for MAPbI_3_ films with and without the TiO_2_ interlayer, indicating improved optoelectronic quality in the presence of TiO_2_. g) Schematic of the test structure used for ion‐migration measurements. Current‐time traces recorded under a constant bias of 40 V for MAPbI_3_ devices h) without and i) with the TiO_2_ interlayer, showing pronounced ion‐driven transient features in the untreated film and a fast, predominantly electronic response for the TiO_2_‐modified film. j) Schematic illustration of the dynamic ion behavior in MAPbI_3_ under an applied electric field: mobile ions migrate to the electrodes during bias, producing an internal ion‐induced potential that opposes the external field and gives rise to transient current peaks at bias application and removal.

To elucidate the beneficial role of the TiO_2_ interlayer, we conducted comprehensive morphological and electronic characterizations. As shown in **Figure** **4**a, when depositing MAPbI_3_ precursor solution onto substrates, the substrates with the TiO_2_ interlayer exhibited significantly smaller contact angles compared to bare substrates, which is crucial for forming uniform and compact perovskite films.^[^ ^21^, ^22^ ^]^ This conclusion was further corroborated by the SEM images in Figure 4b,c. The MAPbI_3_ film without TiO_2_ interlayer displayed relatively small grains (≈100 nm) along with numerous particulates, indicative of poor film quality. In contrast, the film with TiO_2_ interlayer showed larger, more homogeneous grains (≈200 nm) with improved compactness and uniformity. The XRD patterns in Figure 4d reveal a weak PbI_2_ diffraction signal at 13° for the MAPbI_3_ film without TiO_2_ interlayer, indicating a slight incomplete conversion of the perovskite precursor.^[^ ^23^ ^]^ In contrast, the MAPbI_3_ film with TiO_2_ interlayer shows no such signal, and meanwhile, the full width at half maximum of the (110) diffraction peak becomes smaller (Figure S6, Supporting Information), suggesting that the TiO_2_ interlayer effectively enhances the crystallinity of MAPbI_3_ film. Figure 4e,f present the steady‐state and transient PL spectra, respectively. The MAPbI_3_ film with TiO_2_ interlayer exhibits a higher PL intensity at the characteristic peak of 765 nm, suggesting lower defect density.^[^ ^24^ ^]^ Furthermore, the transient PL decay times (τ_1_/τ_2_) are 2.50 and 13.63 ns, respectively, which are longer than those of the MAPbI_3_ film without TiO_2_ interlayer, confirming effective suppression of non‐radiative recombination.^[^ ^25^ ^]^ These results collectively demonstrate that the incorporation of a TiO_2_ interlayer not only improves the crystallographic quality of MAPbI_3_ films but also reduces defect density and suppresses non‐radiative recombination.

To further understand the electronic impact of the interlayer, we performed UPS measurements to determine the energy levels (Figure S7, Supporting Information). We observed two critical changes in the MAPbI_3_ film with the TiO_2_ interlayer. First, both the valence and conduction bands undergo a downward shift of ≈ 0.34 eV. This is likely due to the formation of an interface dipole at the TiO_2_/MAPbI_3_ interface, which alters the local electrostatic potential. Second, the Fermi level shifts upward by 0.15 eV (from ‐4.20 to ‐4.05 eV), moving significantly closer to the conduction band. This upward shift is a clear signature of an n‐doping effect, likely caused by electron transfer from the TiO_2_ interlayer, which is known to have electron‐donating oxygen vacancies. The n‐doping is highly beneficial for N‐type FET performance, as the increased carrier concentration enhances channel conductivity and can passivate electron trap states. Concurrently, the downward shift of the MAPbI_3_ energy bands can reduce the electron injection barrier from the source/drain electrodes (Figure S7d, Supporting Information). These electronic benefits, synergistic with the improved film quality, collectively boost device performance.

Furthermore, we investigated the ion migration in the two films. In MHP FETs, ion migration within the lattice and along grain boundaries becomes particularly pronounced under high electric fields or elevated temperatures. Migrating ions accumulate near the gate, forming local electric fields that partially counteract the externally applied gate field, leading to significant hysteresis or even lost of gate modulation in the transfer characteristics of the FET devices.^[^ ^26^ ^]^ Huang's group previously demonstrated that ions within MHPs migrate under external electric fields (E) exceeding 1 V µm^−1^.^[^ ^27^, ^28^ ^]^ To investigate the ion migration in the perovskite films, we deposited MAPbI_3_ films on substrates, followed by thermal evaporation of Au electrodes with a channel length of 20 µm. A bias voltage of 40 V (E = 2 V/µm) was applied across the Au electrodes, and the current‐voltage relationship over time was recorded to evaluate ion migration (Figure 4g). As shown in Figure 4h, for the MAPbI_3_ device without a TiO_2_ interlayer, the current initially increases upon bias application (t = 2 s), then decreases significantly before slowly increasing again, forming a positive current peak. Upon bias removal (t = 30.4 s), a reverse current peak is observed before the current gradually decays to zero, indicating a dynamic relaxation response. In contrast, for the MAPbI_3_ device with TiO_2_ interlayer, the current increases immediately upon bias application and returns to its original level instantly upon bias removal, demonstrating a rapid voltage‐current response consistent with previous reports (Figure 4i)^.[^ ^29^ ^]^.

The dynamic relaxation behavior of the MAPbI_3_ device can be explained using the schematic shown in Figure 4j. The two gold blocks represent the top electrodes, and the positively and negatively charged spheres represent mobile cations and anions within the MAPbI_3_, respectively. Initially, in the absence of an external electric field (V_bias_ = 0 V), the ions are randomly distributed between the electrodes, maintaining electrical neutrality, and the internal ion‐induced voltage is zero (V_ion_ = 0 V). Upon application of an external bias (V_bias_ > 0 V), ions migrate toward the perovskite/electrode interface under the electric field, inducing an internal ion‐induced voltage opposite to the external bias. The V_ion_ potential partially counteracts the external bias voltage (V_bias_), leading to a reduction in current flow. This corresponds to the initial signal peak observed in Figure 4h. Upon removal of the external bias, the accumulated ions relax back toward equilibrium, producing a transient reverse current that decays as ionic concentration gradients dissipate (Figure 4h)^[^ ^29^, ^30^ ^]^. The positive and negative current peaks observed at bias application and removal are therefore signatures of ion migration in MAPbI_3_; the absence of such peaks in devices with a TiO_2_ interlayer indicates that ion migration is effectively suppressed by the interlayer.

In summary, the TiO_2_ interlayer initiates a cascade of improvements that fundamentally enhance device performance and stability. It first improves wetting of the perovskite precursor, which promotes larger, more uniform grains and improved film compactness, leading to better crystallinity and reduced defect density. Electronically, the interlayer introduces a beneficial n‐doping effect that increases carrier concentration and simultaneously causes a favorable shift in the energy band alignment through interface dipole formation. Finally, the combination of improved crystallinity and a more compact microstructure substantially inhibits ion migration under an applied electric field. Together, these structural, electronic, and ionic improvements account for the markedly superior performance and electrical stability of the MAPbI_3_ FETs incorporating the TiO_2_ interlayer.

### 2.4 Universality of the Interlayer Strategy

**Figure 5:**
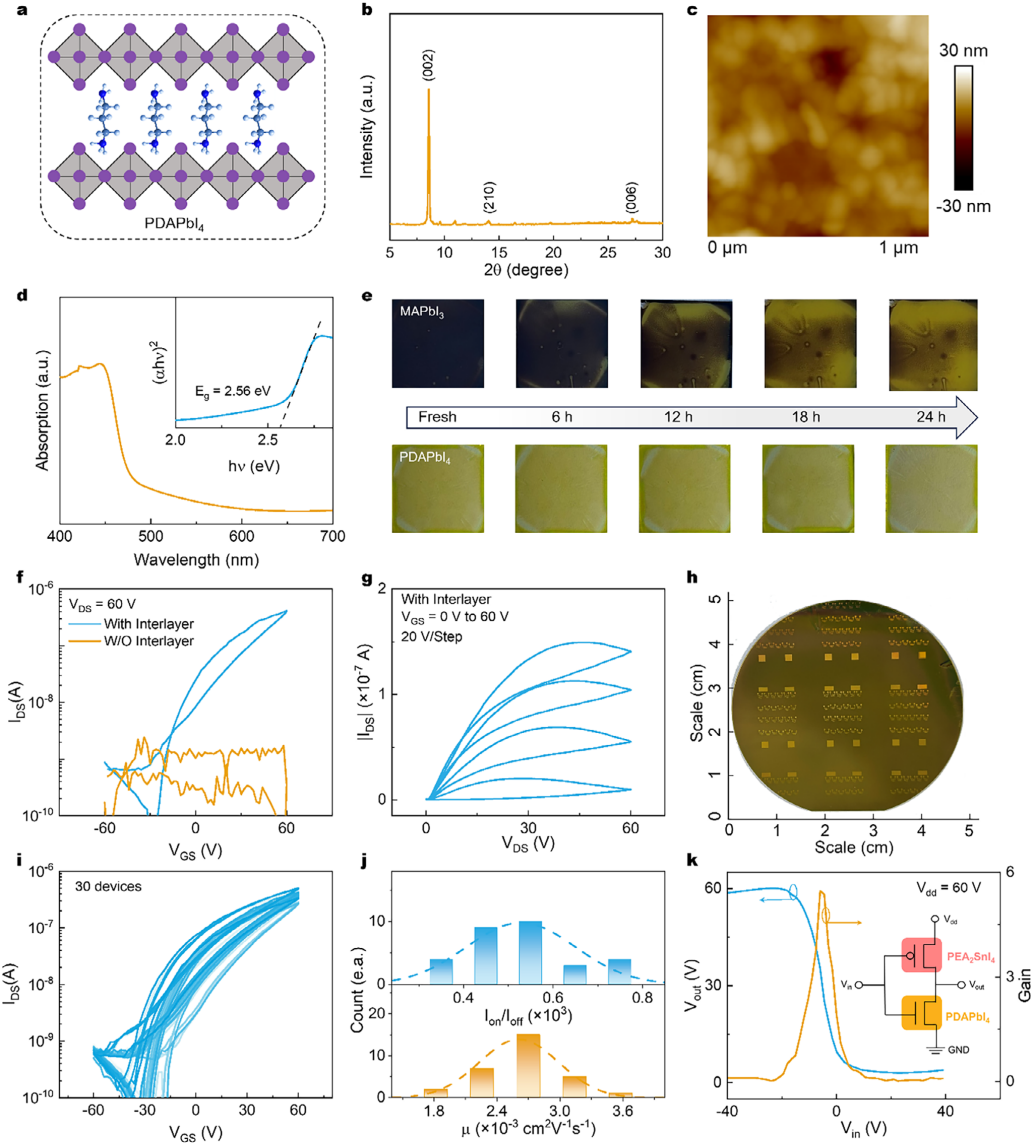
Fabrication and characterization of Dion‐Jacobson phase perovskite FETs. a) The chemical structure and b) XRD pattern of PDAPbI_4_ film. c) AFM image and d) UV–vis absorbance of PDAPbI_4_ film. e) Photographs showing the evolution of MAPbI_3_ and PDAPbI_4_ films in the atmospheric environment with time. f) Transfer and g) output characteristics of PDAPbI_4_ FET. h) The photo of the 2‐inch PDAPbI_4_ FET arrays. i) Transfer curves of 30 randomly selected devices from a large‐area PDAPbI_4_ FET array prepared. j) Statistical distribution of the mobility and on/off ratio of the 30‐device array. k) The output voltage and gain as a function of input voltage for the all‐perovskite inverter.

After confirming the role of the ultrathin TiO_2_ interlayer, we explored the universality of this strategy. Recent advances in N‐type MHP FETs have predominantly focused on 3D MHPs and 2D Ruddlesden‐Popper (RP) phases^[^ ^31^, ^32^ ^]^; N‐type DJ phase MHP FETs remain unreported despite DJ phases offering enhanced environmental stability relative to 3D MHPs and superior out‐of‐plane charge transport compared with 2D RP perovskites.^[^ ^24^ ^]^ Motivated by our success with MAPbI_3_, we extended the interlayer approach to 2D DJ‐phase perovskite PDAPbI_4_ (**Figure** **5**a) and obtained room‐temperature functional FETs.

Figure 5b shows the XRD of solution‐processed PDAPbI_4_ films, exhibiting sharp reflections at 8.6°, 14.1°, and 27.4° assignable to the (002), (210), and (006) planes, respectively.^[^ ^33^ ^]^ Atomic force microscopy (AFM) revealed uniformly distributed grains of ≈100 nm with dense, continuous morphology (Figure 5c). Optical absorption spectra showed a sharp edge at 450 nm, corresponding to a bandgap of 2.56 eV (Figure 5d). Notably, PDAPbI_4_ demonstrates significantly superior environmental stability compared to MAPbI_3_. As evidenced in Figure 5e, while MAPbI_3_ films underwent complete degradation (manifested as black‐to‐yellow transition) within 24 h under ambient conditions, PDAPbI_4_ maintained its colors throughout the same period. This enhanced stability originates from the hydrophobic organic cations in the DJ structure that protect the inorganic sheets from moisture and other environmental agents.^[^ ^24^, ^34^ ^]^

Using identical device fabrication protocols, we converted these films into FETs. PDAPbI_4_ devices deposited on bare substrates could not work, exhibiting source‐drain currents below 10^−9^ A throughout gate voltage sweeps (±60 V). Remarkably, incorporation of the ultrathin TiO_2_ interlayer enabled robust N‐type FETs, with an on/off ratio greater than 10^3^ (Figure 5f) and output curves showing clear gate modulation (Figure 5g). Comparative stability tests revealed PDAPbI_4_ FETs maintained performance after 20 days in an Ar‐filled glovebox, while MAPbI_3_ FETs degraded substantially within just 6 days, highlighting the superior potential of 2D DJ‐phase perovskites (Figure S8, Supporting Information).

To demonstrate scalability and device uniformity, we fabricated a 2‐inch wafer‐scale PDAPbI_4_ FET array using our anti‐solvent‐free, single‐step spin‐coating approach (Figure 5h). Statistical analysis of 30 devices showed uniform performance: on‐state currents and threshold voltages remained stable, while mobility and on/off ratios followed normal distributions with peak values reaching 3.6 × 10^−3^ cm^2^V^−1^s^−1^ and 7.5 × 10^2^, respectively (Figure 5i,j). The consistent yield and stability of these N‐type devices provide a practical basis for realizing large‐area electronics.

As a proof‐of‐concept for integrated logic, we combined our TiO_2_‐enabled N‐type PDAPbI_4_ FETs with P‐type PEA_2_SnI_4_ FETs (Figure S9, Supporting Information) to construct an all‐MHP inverter. The inverter shows clear voltage inversion and a peak gain approaching 6 (Figure 5k), demonstrating the feasibility of perovskite‐only logic elements.

Finally, we extended the interlayer strategy to additional DJ‐phase perovskites by alloying MA^+^ with PDA ^2^ ^+^ to form PDAMAPb_2_I_7_ and PDAMA_2_Pb_3_I_10_. Films processed without the TiO_2_ interlayer again yielded negligible transistor currents (I_DS_ < 10^−9^ A) under gate voltage sweeps (Figure S10, Supporting Information). In stark contrast, devices with the interlayer demonstrated standard N‐type FETs behavior, featuring a near‐zero threshold voltage, an on/off ratio of 10^3^, and well‐modulated output characteristics (Figure S11, Supporting Information). These results confirm that the ultrathin TiO_2_ interlayer is an effective, generalizable strategy to enable and stabilize N‐type transport across a range of Pb‐based perovskites.

## 3 Conclusion

In conclusion, this work introduces a simple yet powerful interlayer strategy that fundamentally addresses critical challenges in Pb‐based perovskite FETs. By strategically introducing an ultrathin TiO_2_ interlayer, we have demonstrated a reproducible approach to fabricating high‐performance N‐type MHP FETs that overcomes longstanding limitations such as poor film quality, high defect densities, and significant ion migration. Our comprehensive investigations reveal that the TiO_2_ interlayer enhances precursor wetting, promotes larger and more uniform grain formation, reduces defect density, and effectively suppresses non‐radiative recombination and ion migration. Beyond MAPbI_3_, we have successfully extended this strategy to 2D Dion‐Jacobson phase perovskites, including PDAPbI_4_ and its derivatives, highlighting the universality of our approach. This work not only provides a straightforward fabrication method for high‐performance Pb‐based perovskite FETs but also offers fundamental insights into improving the electronic properties of metal halide perovskite semiconductors.

## 4 Experimental Section

### Materials

Methylammonium iodide (MAI, ≥ 99.5%), propane‐1,3‐diammonium (II) iodide (PDAI_2_, ≥ 99.99%), and Lead (II) iodide (PbI_2_, ≥ 99.99%) were purchased from Xi'an Polymer Light Technology Corp. Poly(vinylidenefluoridetrifluoroethylene) (P(VDF‐TrFE), 70:30 mol%) and poly(vinylidene fluoride‐co‐hexafluoropropylene) (PVDF‐HFP) were purchased from Piezotech. Lead acetate trihydrate (PbAc_2_, 99.998%), n‐butyl Acetate (nBA, 99.5%), 2‐butanone (MEK, 98%), and N, N‐dimethylformamide (DMF, anhydrous, 99.8%) were bought from Sigma‐Aldrich. Cytop solution was prepared by adding Cytop solvent into Cytop with a volume ratio of 1:3. TiO_2_ solution was obtained by mixing TiO_2_ colloidal and anhydrous ethanolin. All materials were used as received without any additional purification.

### Preparation of MAPbI_3_ Films

For the preparation of MAPbI_3_ precursor solutions, two distinct methods were employed. In the PbI_2_ method, MAPbI_3_ was synthesized from a precursor solution containing 0.75 M PbI_2_ and MAI in a 1:1 molar ratio dissolved in DMF. In the PbAc_2_ method, MAPbI_3_ was formed by mixing 0.75 M PbAc_2_ and MAI in a 1:3 molar ratio in DMF. Both precursor solutions were heated at 45 °C for 10 h within an Ar‐filled glovebox to ensure complete dissolution. Prior to use, the solutions were filtered through 0.45 µm PTFE filters to remove any undissolved particulates. For film fabrication, the PbI_2_ method involved spin‐coating the precursor solution at 4000 rpm for 30 s. During the spin‐coating process, a droplet of chlorobenzene (CB) was applied to the sample surface at the 5‐s mark to promote uniform crystallization. In the PbAc_2_ method, the precursor solution was spin‐coated directly onto the substrates at 4000 rpm for 30 s without the addition of chlorobenzene. All perovskite films were subsequently annealed at 150 °C for 2 min in the Ar‐filled glovebox to enhance crystallinity and film quality.

### Preparation of PDAMA_n‐1_Pb_n_I_3n+1_ Films

The preparation process of PDAMA_n‐1_Pb_n_I_3n+1_ (n = 1–3) is as follows: PbI_2_, MAI, and PDAI_2_ are weighed according to a specific molar ratio and dissolved in DMSO solvent to prepare a precursor solution with a concentration of 0.05 m. The spin‐coating process is carried out at 4000 rpm for 30 s, and no anti‐solvent is added during the spin‐coating process. Finally, the film is annealed at 150 °C for 1 min to complete crystallization and film optimization.

### Fabrication of FET

For the BGBC devices, Cr/Au (3/32 nm) electrodes were first pre‐patterned on Si/SiO_2_ substrates using photolithography. These substrates were then sequentially cleaned by ultrasonication in ultrapure water, acetone, and isopropanol for 5 min each, followed by UV/ozone treatment for 15 min. A TiO_2_ precursor solution was spin‐coated onto the substrates at 4000 rpm for 30 s and annealed at 150 °C for 30 min in ambient conditions. Subsequently, the perovskite film was directly deposited via spin‐coating inside a glovebox. For the MAPbI_3_ TGBC devices, the substrate used for fabrication was replaced with glass slides. The preparation methods for TiO_2_ and MAPbI_3_ remained the same as those for the BGBC devices. Additionally, a dielectric layer was spin‐coated on top of the MAPbI_3_ film. The channel length and width of the device are 20 and 1000 µm, respectively. Finally, a 50 nm Al gate electrode was deposited by thermal evaporation through a shadow mask to complete the device fabrication. For the dielectric layers, either P(VDF‐TrFE) (70 mg mL^−1^, dissolved in n‐butyl acetate, nBA) or PVDF‐HFP (100 mg mL^−1^, dissolved in methyl ethyl ketone, MEK) was spin‐coated onto the perovskite film using a two‐step program: the first step at 500 rpm for 3 s and the second step at 4000 rpm for 20 s. Post‐annealing at 90 °C for 1 h was performed to enhance the film quality. The spin‐coating processes for Cytop followed conventional protocols. The device fabrication of the PDAMA_n‐1_Pb_n_I_3n+1_ FET with the BGBC structure is the same as the above. The channel length and width of the device used are 60 and 1000 µm, respectively.

### Characterization of Film

The film morphology was measured by SEM (TESCAN MIRA3) and atomic force microscopy (AFM) (Bioscope system). The X‐ray diffraction (XRD) patterns were recorded by D/max 2550 (Rigaku) under Cu Kα (λ = 1.5406 Å) irradiation in the ambient atmosphere. PL spectra were recorded by a Thermo Scientific Lumina. The ultraviolet‐visible‐near‐infrared (UV‐vis‐NIR) absorption spectra of perovskite films were characterized with UV‐3600 PLUS (Shimadzu).

### Measurement of FET

Electrical properties of FET devices were performed with a Keysight B2912A Precision Sources through a probe station in an Ar glovebox. All measurement of FETs is carried out under dark conditions. It should be noted that all the measurement procedures were measured in DC mode rather than pulse mode.

### Characterization of MAPbI_3_ Phototransistor

During light detection, the light source was placed in a dark glovebox to avoid influence by ambient light. The light at 660 nm was emitted using commercial light sources. The intensity of the incident light at different wavelengths was calibrated by a commercial silicon photodetector. The responsivity (R) is defined as $R=\;\frac{I_{\mathrm{light}}-\;I_{\mathrm{dark}}}{P\times S}$, where I_light_ and I_dark_ represent the current under illumination and in dark conditions, respectively. *P* is the incident light power intensity, and *S* is the effective active area of the MAPbI_3_ FET device. If one assumes that the dark current is mainly originates from shot noise of dark current (I_dark_), the specific detectivity (D*) can be written as: D^*^ = $\frac{R}{\sqrt{2q(\frac{I_{\mathrm{dark}}}{S})}}$.

### Statistical Analysis

The performance data of the perovskite FETs for n devices (n = 30) were summarized and presented as a distribution graph without pre‐processing. The mean or standard deviation values were not calculated. The data processing was performed by Origin software.

## Conflict of Interest

The authors declare no conflict of interest.

## Supporting information



Supporting Information

## Data Availability

The data that support the findings of this study are available from the corresponding author upon reasonable request.
