# Supplementary material for: Enabling Robust N‐Type Perovskite Field‐Effect Transistors Through an TiO2 Interlayer Strategy
Source: Adv Sci (Weinh). 2025 Oct 7;12(48):e16610. doi: 10.1002/advs.202516610 (PMC12752618; doi:10.1002/advs.202516610)
Supplement: Supplementary file 1 — Supporting Information [file ADVS-12-e16610-s001.docx]

**Supplementary Information**

**Enabling Robust N-Type Perovskite Field-Effect Transistors through an TiO_2_ Interlayer Strategy**

Jiangnan Xia^1,2^, Xincan Qiu^3^, Ping-An Chen^1^, Yu Liu^3^, Jiaqi Ding^2^, Yu Zhang^1^, Huan Wei^2^, Zhenqi Gong^1^, Chengyuan Peng^1^, Wenpei Shi^1^, Shuanglong Wang^5^, Chen Chen^4*^, Yuanyuan Hu^1,2,3*^

^1^International Science and Technology Innovation Cooperation Base for Advanced Display Technologies of Hunan Province, College of Semiconductors (College of Integrated Circuits), Hunan University, Changsha 410082, China.

^2^Key Laboratory for Micro/Nano Optoelectronic Devices of Ministry of Education, School of Physics and Electronics, Hunan University, Changsha 410082, China.

^3^Key Laboratory of Hunan Province for 3D Scene Visualization and Intelligence Education, School of Electronic Information, Hunan First Normal University, Changsha 410205, China.

^4^Science and Technology on Advanced Ceramic Fibers and Composites Laboratory, College of Aerospace Science and Engineering, National University of Defense Technology, Changsha 410000, China.

^5^Department of Applied Physics, The Hong Kong Polytechnic University, Hong Kong 999077, China.

Email of the corresponding author: [angela_chen@nudt.edu.cn](mailto:angela_chen@nudt.edu.cn); [yhu@hnu.edu.cn](mailto:yhu@hnu.edu.cn)

**1. Optimization of MAPbI_3_ film fabrication processes**

**
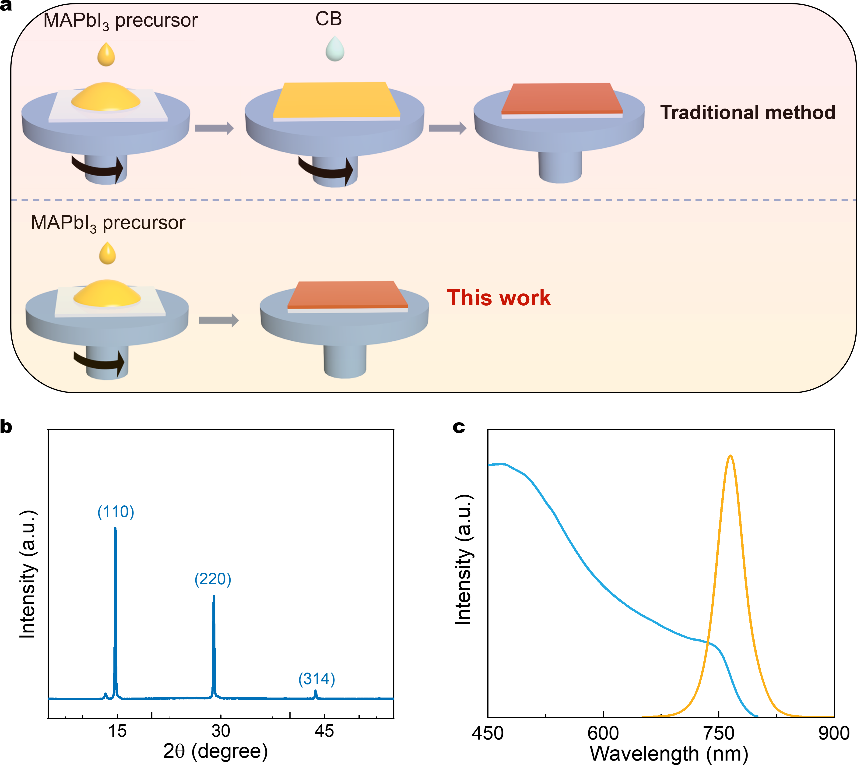
**

**Figure S1.** **Optimization of MAPbI_3_ fabrication process**. Optimization of MAPbI_3_ fabrication process. (a) Schematic illustration of the traditional fabrication process of MAPbI_3_ films and the process developed in this work. (b) XRD patterns of MAPbI_3_ film. (c) Absorption and PL spectra of MAPbI_3_ thin film.

Conventional solution processing of MAPbI_3_ thin films typically requires anti-solvent treatment (e.g., chlorobenzene or toluene) to induce crystallization. The reproducibility of both the films and devices is highly sensitive to multiple anti-solvent parameters, including the processing window, volume, and dropping timing. This makes our developed anti-solvent-free, one-step spin-coating method particularly valuable. When combined with high-temperature, short-duration annealing (effectively removing byproducts), this simplified process significantly improves the fabrication reproducibility of MAPbI_3_ films.

**2. Top-gate MAPbI_3_ FET properties used TiO_2_ interlayer**


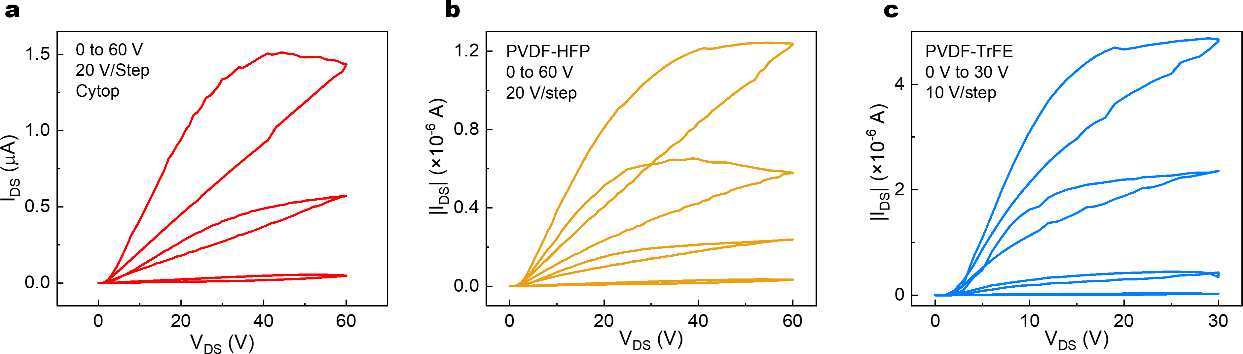


**Figure S2.** Output characteristics of TGBC MAPbI_3_ FETs using (a) Cytop, (b) PVDF-HFP, and (c) PVDF-TrFE as dielectric layers, respectively.


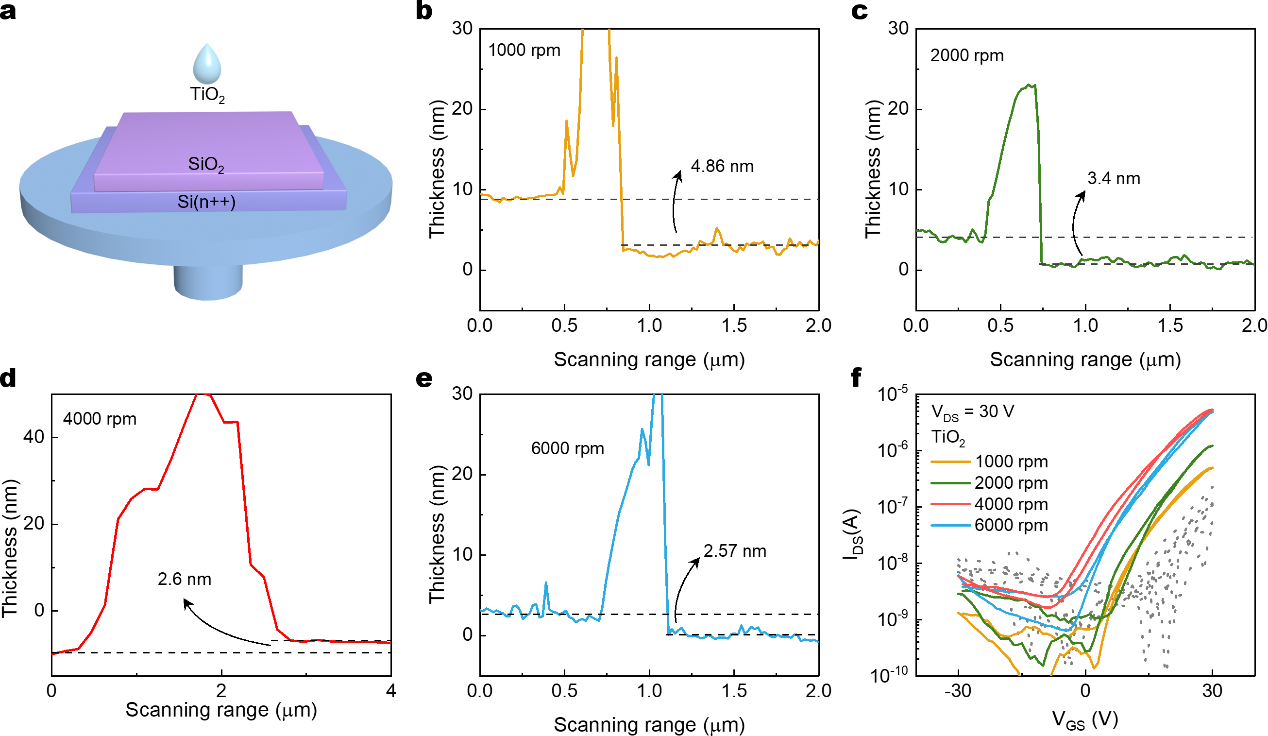


**Figure S3. Effects of different TiO_2_ thicknesses on the performance of MAPbI_3_ FET devices.** (a) Schematic diagram of the preparation process of TiO_2_ thin films with different thicknesses. The thicknesses of TiO_2_ thin films prepared at spin-coating speeds of (b) 1000 rpm, (c) 2000 rpm, (d) 4000 rpm, and (e) 6000 rpm respectively. (f) Transfer characteristic curves of MAPbI_3_ FETs at different thicknesses.

Our comprehensive study systematically investigated this relationship through controlled fabrication of TiO_2_ films using spin-coating at varying rotational speeds (1000-6000 rpm). High-resolution thickness measurements (**Figures S3a-e**) reveal a clear trend: increasing spin speed from 1000 rpm to 6000 rpm reduces the TiO_2_ thickness from 4.86 nm to 2.57 nm, with the rate of thickness reduction diminishing significantly beyond 4000 rpm (Δd ≈ 0.03 nm between 4000 rpm and 6000 rpm), suggesting a practical lower limit for thickness control using this fabrication method.

The corresponding electrical characterization of MAPbI_3_ FET devices (**Figure S3f**) demonstrates two important thickness-dependent effects: First, we observe a progressive enhancement in on-state current (𝐼_on_) as TiO_2_ thickness decreases, eventually reaching saturation at the thinnest layers. Second, systematic negative shifts in threshold voltage (𝑉_th_) correlate with reduced TiO_2_ thickness. These phenomena collectively suggest that the TiO_2_ interlayer thickness critically modulates the contact barrier at the MAPbI_3_/source-drain electrode interface, thereby influencing both carrier injection efficiency and overall device performance. The saturation of 𝐼_on_ improvement beyond 4000 rpm correlates well with the observed thickness saturation, reinforcing the direct connection between interfacial engineering and device characteristics.


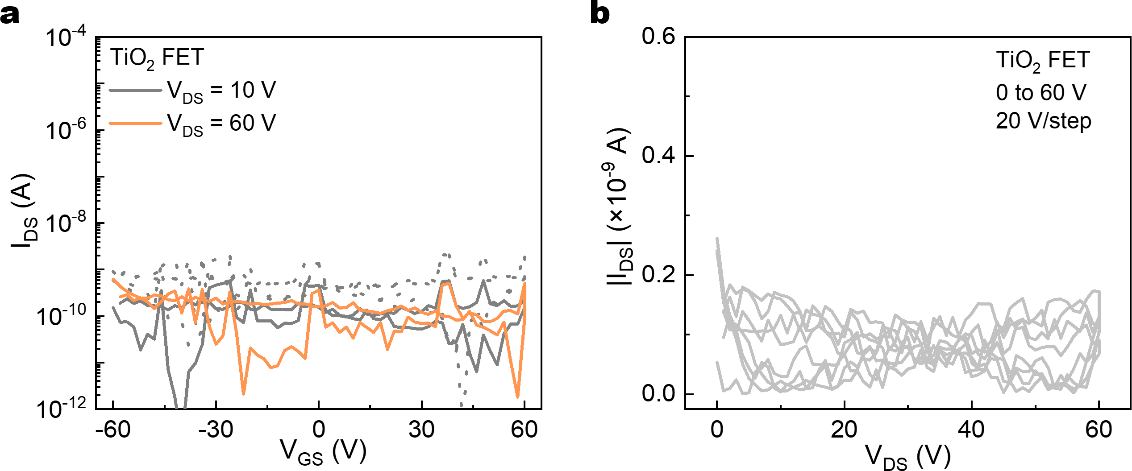


**Figure S4.** **Intrinsic FET performance of TiO_2_**. (a) Transfer characteristic curve and (b) output characteristic curve of the TiO_2_ FET.

Control experiments with BGBC-structured TiO_2_ FETs confirmed the absence of measurable FETs characteristics, definitively excluding TiO_2_ as a contributing channel material. This evidence conclusively demonstrates that the observed performance enhancement in MAPbI_3_ FETs originates solely from the interfacial modification by the TiO_2_ interlayer, rather than parallel conduction pathways.


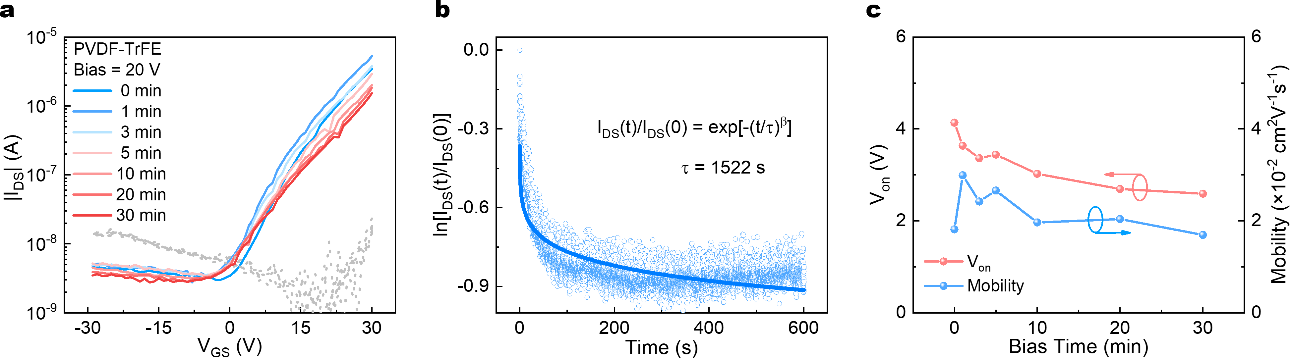


**Figure S5. Bias stability test of the MAPbI_3_ FET** **with TiO_2_ interlayer**. (a) The transfer characteristics of the PVDF-TrFE/MAPbI_3_ devices are recorded at different times during the bias-stress conditions (V_GS_ = 20 V, V_DS_ = 0 V). (b) The decay of on-state current with bias-stressing time (V_GS_ = 20 V and V_DS_ = 5 V). The fitting of the date by the equation and the resultant characteristic time is indicated. (c) Schematic curves of on-state voltage and mobility with bias-stress.

We tested their bias stability by applying a 20 V pre-bias to the gate before measuring the transfer characteristics. As shown in **Figure S5a**, the on-state voltage remained nearly unchanged, with only a slight decrease in the on-state current as the pre-bias duration increased. Additionally, we measured the channel current under a gate voltage of 20 V and a source-drain voltage of 5 V (**Figure S5b**), fitting the data with an exponential function, which yielded a time constant (τ) of 1522 s. **Figure S5c** also shows the on-state voltage and mobility of TGBC MAPbI_3_ FET device as a function of pre-bias time, both of which remained stable.

**3. The influence of TiO_2_ on MAPbI_3_ thin films.**


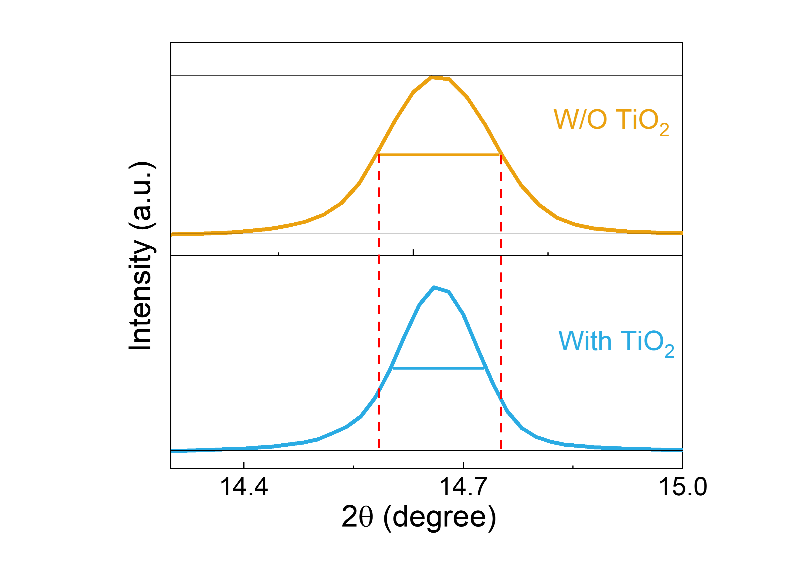


**Figure S6.** Comparison of the full width at half maximum (FWHM) of the diffraction peaks of the (110) crystal plane of MAPbI_3_ with and without an interlayer.


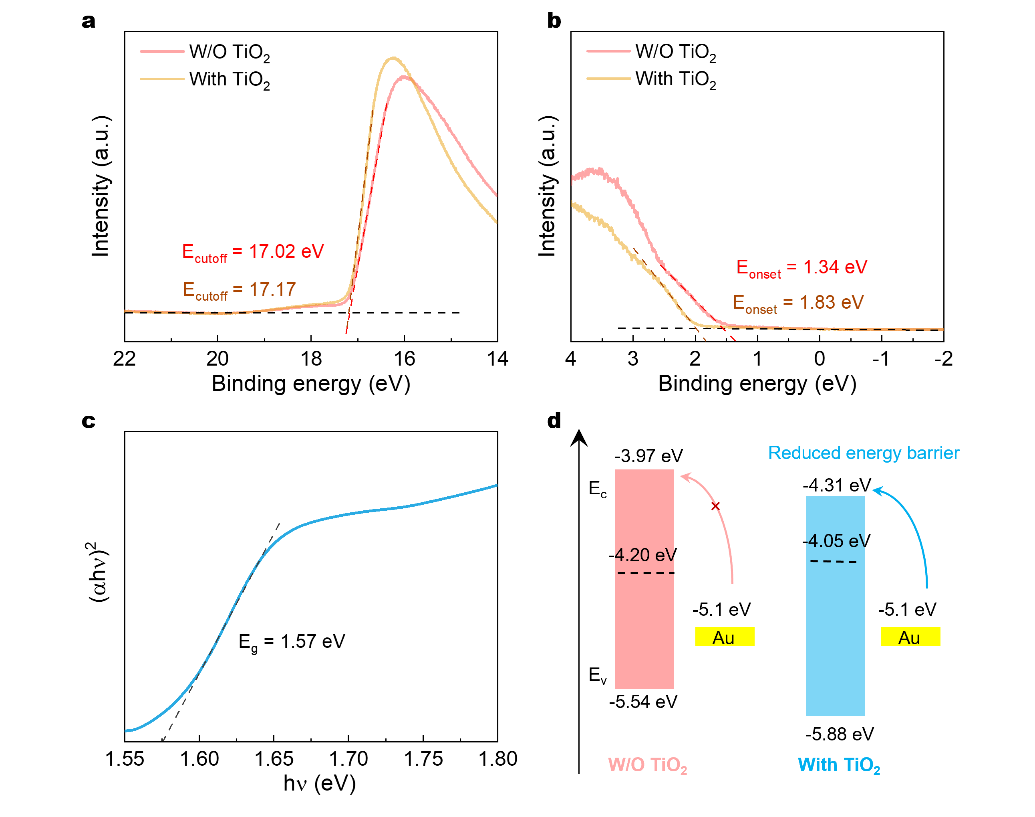


**Figure S7.** **UPS characterizations of MAPbI_3_ thin films with and without TiO_2_ interlayers.**

As shown in **Figure S7**, UPS measurements were performed to analyze the energy levels of MAPbI_3_ with and without the TiO_2_ interlayer. The results reveal that pristine MAPbI_3_ exhibits valence band (VB) and conduction band (CB) positions at -5.54 eV and -3.97 eV, respectively. In contrast, MAPbI_3_ with the TiO_2_ interlayer demonstrates shifted energy levels, with VB at -5.88 eV and CB at -4.31 eV. The downward shift of the MAPbI_3_ bands suggests the dipole is oriented with its positive side pointing away from the interface (into the MAPbI_3_) and its negative side pointing toward the TiO_2_ substrate. This doward shift of bands effectively reduces the Schottky barrier, facilitating more efficient electron injection.

Notably, the Fermi level of MAPbI_3_ shifts toward the conduction band when incorporating the TiO_2_ interlayer, resembling an n-doping effect. The doping mechanism of TiO_2_ is not clear yet, which deserves further investigation in future studies. Nevertheless, the n-doping effect beneficial for enhancing N-type FET performance as the induced carriers can fill traps, functioning as trap passivation. These comprehensive results demonstrate that the TiO_2_ interlayer plays a critical role in optimizing the performance of MAPbI_3_ FETs.

**4. Storage stability of the MAPbI_3_ and PDAPbI_4_ FET.**

**
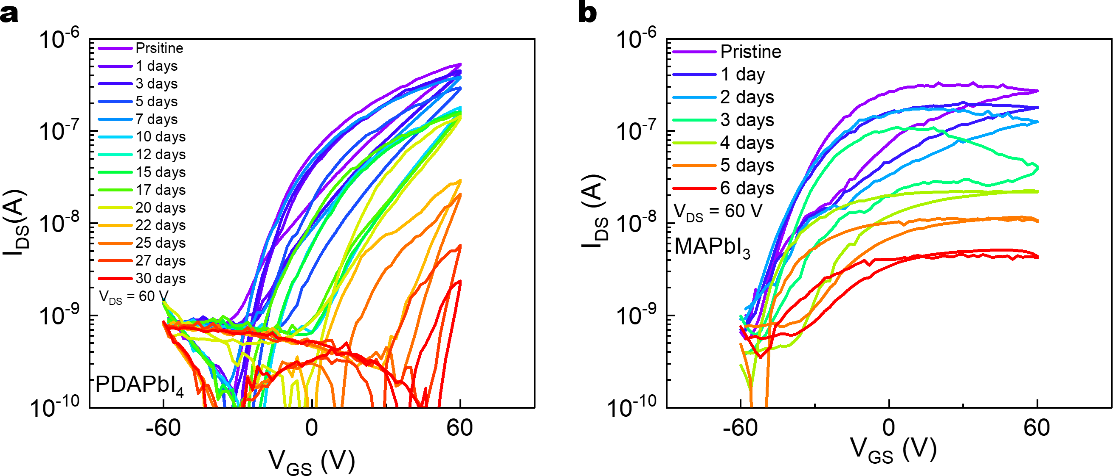
**

**Figure S8.** **Storage stability of the MAPbI_3_ and PDAPbI_4_ FET**. Evolution of transfer characteristics of (a) PDAPbI_4_ and (b) MAPbI_3_ FETs stored in glovebox.

When comparing the stability of PDAPbI_4_ and MAPbI_3_ FETs, all BGBC-structured devices were evaluated under identical argon-filled glovebox conditions. The PDAPbI_4_ FETs exhibited both superior initial performance metrics and enhanced operational stability compared to their MAPbI_3_ counterparts.

**5. Fabrication of all-perovskite inverters.**


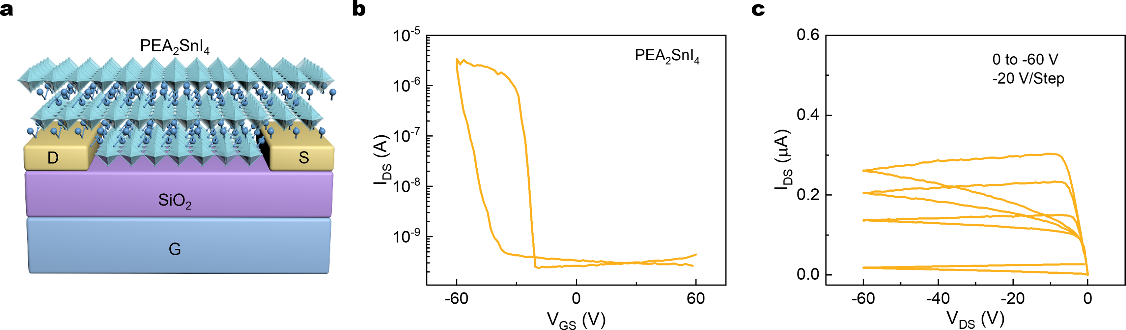


**Figure S9.** **Performance of the P-type perovskite FET device in the inverter circuit**. (a) Schematic diagram of the BGBC PEA_2_SnI_4_ FET. (b) Transfer characteristic curve and (c) output characteristic curve of the PEA_2_SnI_4_ BGBC device.

**6. The universality of the interlayer strategy.**


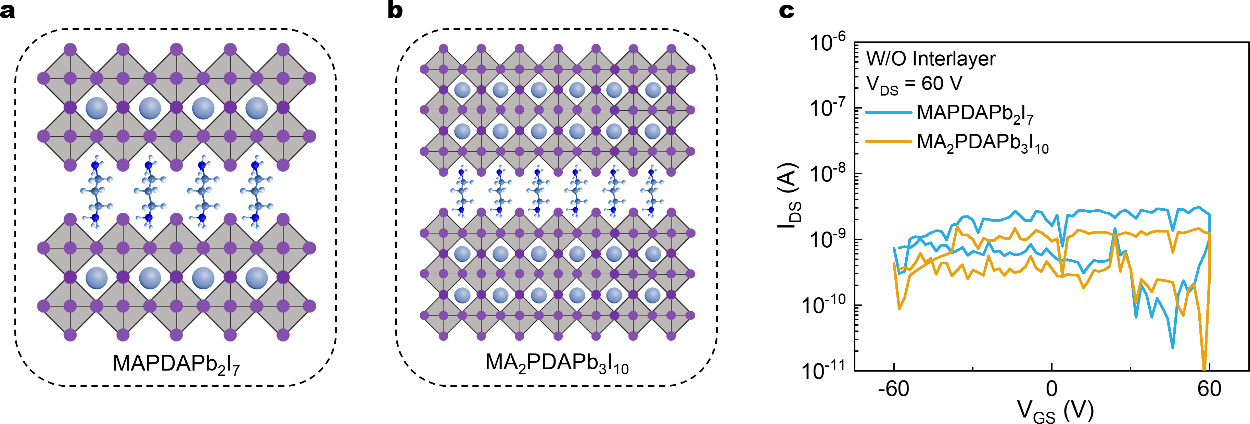


**Figure S10.** **The performance of MA_n-1_PDAPb_n_I_3n+1_ FETs without interlayer.** (a) The chemical structure of MAPDAPb_2_I_7_ film. (b) The chemical structure of MA_2_PDAPb_3_I_10_ film. (c) Transfer characteristic curve of the PDAMA_n-1_Pb_n_I_3n+1_ (n=2 and n=3) BGBC FET device without interlayer.


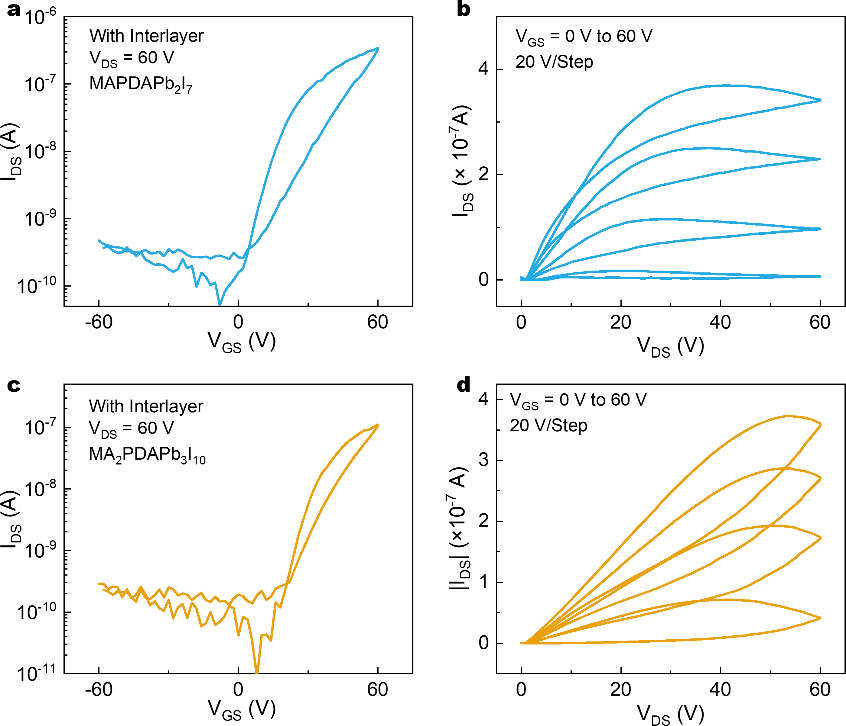


**Figure S11.** **The performance of MA_n-1_PDAPb_n_I_3n+1_ FETs with interlayer.** (a) Transfer and (b) output characteristics of MAPDAPb_2_I_7_ FET. (c) Transfer and (d) output characteristics of MA_2_PDAPb_3_I_10_ FET.
